# Prevalence of HPV 16 and HPV 18 Lineages in Galicia, Spain

**DOI:** 10.1371/journal.pone.0104678

**Published:** 2014-08-11

**Authors:** Sonia Pérez, Ana Cid, Amparo Iñarrea, Mónica Pato, María José Lamas, Bárbara Couso, Margarita Gil, María Jesús Álvarez, Sonia Rey, Isabel López-Miragaya, Santiago Melón, María de Oña

**Affiliations:** 1 Department of Microbiology, University Hospital of Vigo, Vigo, Spain; 2 Department of Microbiology, University Hospital of Ourense, Ourense, Spain; 3 Department of Obstetrics and Gynecology, University Hospital of Vigo, Vigo, Spain; 4 Department of Obstetrics and Gynecology, University Hospital of Ourense, Ourense, Spain; 5 Department of Pathology, University Hospital of Vigo, Vigo, Spain; 6 Department of Virology, University Hospital of Asturias, Oviedo, Spain; Albert Einstein College of Medicine, United States of America

## Abstract

Genetic variants of human papillomavirus types 16 and 18 (HPV16/18) could differ in their cancer risk. We studied the prevalence and association with high-grade cervical lesions of different HPV16/18 variant lineages in a case-control study including 217 cases (cervical intraepithelial neoplasia grade 2 or grade 3 or worse: CIN2 or CIN3+) and 116 controls (no CIN2 or CIN3+ in two-year follow-up). HPV lineages were determined by sequencing the long control region (LCR) and the E6 gene. Phylogenetic analysis of HPV16 confirmed that isolates clustered into previously described lineages: A (260, 87.5%), B (4, 1.3%), C (8, 2.7%), and D (25, 8.4%). Lineage D/lineage A strains were, respectively, detected in 4/82 control patients, 19/126 CIN3+ cases (OR = 3.1, 95%CI: 1.0–12.9, p = 0.04), 6/1 glandular high-grade lesions (OR = 123, 95%CI: 9.7–5713.6, p<0.0001), and 4/5 invasive lesions (OR = 16.4, 95%CI: 2.2–113.7, p = 0.002). HPV18 clustered in lineages A (32, 88.9%) and B (4, 11.1%). Lineage B/lineage A strains were respectively detected in 1/23 control patients and 2/5 CIN3+ cases (OR = 9.2, 95%CI: 0.4–565.4, p = 0.12). In conclusion, lineages A of HPV16/18 were predominant in Spain. Lineage D of HPV16 was associated with increased risk for CIN3+, glandular high-grade lesions, and invasive lesions compared with lineage A. Lineage B of HPV18 may be associated with increased risk for CIN3+ compared with lineage A, but the association was not significant. Large well-designed studies are needed before the application of HPV lineage detection in clinical settings.

## Introduction

The causal association of high-risk types of human papillomavirus (HR-HPV) with cervical cancer has been well established [1]. The HR-HPV types are highly prevalent in the general population, but the reasons that favor only a small proportion of infections to persist and progress to cancerous lesions are still poorly understood. The association between HPV16 or HPV18 and cervical cancer is precisely related to the DNA composition of their specific genome (odds ratio higher than 300 and higher than 150, respectively) [2].

HPVs contain a circular double-stranded DNA genome of approximately 7.9 kb that consists of eight protein-coding genes divided in an early region (E6, E7, E1, E2, E4, E5) and a late region (L2, L1) and two noncoding regions (the noncoding region [NCR] and the long control region [LCR]). Since complete HPV genomes were available and phylogenetic analysis was applied to the study of HPV, variant lineages would be defined by an approximately 1.0% difference between full genomes of the same HPV type and variant sublineages by a 0.5–1.0% difference between lineages. HPV lineage distribution is related to geographic or race distribution [3]. Nomenclature of these lineages and sublineages has been updated recently [4].

For HPV16, phylogenetic analysis confirmed the description of four lineages [3]: A (previously called European-Asian, EAS), B (African 1, Afr1), C (African 2, Afr2), and D (North-American/Asian-American, NA/AA). Furthermore, nine sublineages have been described: A1, A2, A3 (European, E), A4 (Asian, As), B1 (Afr1a), B2 (Afr1b), D1 (NA), D2 (AA1), and D3 (AA2) [4].

In case of HPV18, three lineages have been described [5]: A (that includes previously called Asian-American and European lineages, AA and E, respectively), B, and C (these two include previous African, Af). Eight sublineages have also been described for this type [4].

Some non-lineage-specific single nucleotide polymorphisms (SNPs) appear independently of lineage, like 350G polymorphism in the E6 region that has been reported to be frequent in the E sublineages (A1, A2, A3) and in AA (D2, D3) sublineages of HPV16.

Nucleotide changes in HPV16 and HPV18 lineages may interfere with the viral oncogenic potential, and it is unknown whether any modification in the amino acids of the viral capsid may affect the efficacy of vaccination.

Several investigations have suggested the influence of viral genetic variation between HPV16 and HPV18 lineages in viral persistence and the development of cervical cancer [6]–[15].

The aim of the present study was to know the prevalence and association with high-grade cervical lesions of different HPV16/18 variant lineages in Galicia, Spain.

## Materials and Methods

### Ethics Statement

This study received approval from the Ethics Committee of Clinical Investigation of Galicia (Santiago de Compostela, Spain). All study participants were informed about the purpose of the survey and were asked to sign a consent form before taking part in the study. Confidentiality was ensured during data collection and subsequent publication of the results.

### Patients and Follow-Up

**Figure 1:**
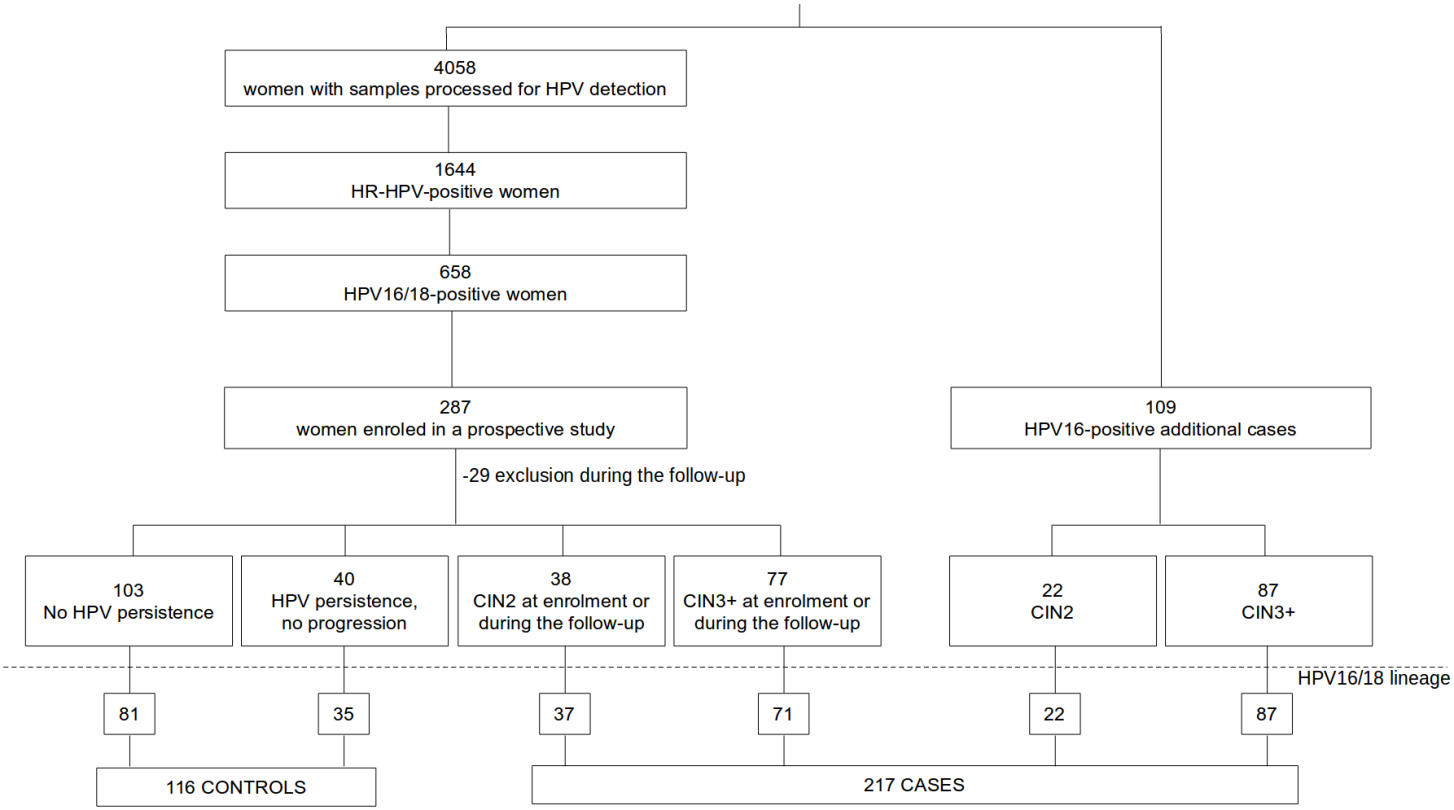
Study population. CIN2: Cervical intraepithelial neoplasia grade 2. CIN3+ includes CIN grade 3-carcinoma *in* *situ*, invasive squamous cell carcinoma, adenocarcinoma *in* *situ*, and adenocarcinoma. Controls for the analysis were women without progression to CIN2 or CIN3+ during the two-year follow-up of the prospective study of HPV16/18-positive women. Cases were women with histological diagnosis of HPV16/18-positive CIN2 or CIN3+ at enrolment or during the two-year follow-up of the prospective study. Enrolment of the prospective study: from 2009 to 2010. Additional cases were HPV16-positive CIN2 or CIN3+ cases collected from 2006 to 2008 and 2011 to 2012.

Women attending the Department of Obstetrics and Gynecology of two university hospitals in Galicia, Spain (Vigo and Ourense), for cervical cancer screening (2006–2012) positive for HPV16 (n = 333) or HPV18 (n = 63) were included in a case-control study (figure 1).

Between 2009 and 2010, samples from 4058 women were processed for HPV detection in the Microbiology Departments of these hospitals. The presence of HR-HPV was detected in 1644 women. HPV16/18-positive women were offered to be enroled in a prospective study. Two hundred and eighty-seven out of 658 HPV16/18-positive women were included. Patients were followed for two years with cytology and virological detection every six months to assess viral persistence and progression of the cervical lesions. Colposcopy was performed in case of abnormal cytology, and biopsy was taken in case of abnormal colposcopy. Follow-up was discontinued during pregnancy and continued four months after delivery. Patients with intraepithelial neoplasia grade 2 (CIN2) or CIN grade 3 diagnosis or worse (CIN3+) at any time during the study received treatment (conization, hysterectomy or radiotherapy) with the corresponding cytological and virological monitoring after treatment. Main exclusion conditions were conization in the previous 12 months, missing follow-up, or treatment for CIN grade 1.

They were designated as cases (histological diagnosis of CIN2 or CIN3+) or controls (no viral persistence or no progression to CIN2 or CIN3+ in two-year follow-up).

Besides, 109 HPV16-positive cases diagnosed from 2006 to 2008 and 2011 to 2012 in the same department of the University Hospital of Vigo were included.

### Epidemiological Data

Data for age at enrolment and age at the first worst histological diagnosis of CIN2 or CIN3+ were collected.

Patients answered a voluntary self-questionnaire composed of questions regarding sociodemographic variables such as race, smoking, and hormonal contraceptives use and questions regarding sexual behavior like age at first sexual contact in order to calculate years since first sexual contact at enrolment.

All women were suggested to be tested to know their HIV serological status. This data was collected when available.

The study was approved by the Ethics Committee of the Clinical Investigation of Galicia (Santiago de Compostela, Spain).

### Cytological and Histological Diagnoses

Cytological examinations of Pap smear were reported using the 2001 Bethesda Reporting System [16].

Cervical biopsy specimens were sampled under a colposcopic guide. Additional biopsies were studied in case of conization or hysterectomy. The worst histological diagnosis throughout the clinical course of the patient was considered in the classification of the lesion.

Diagnoses of moderate dysplasia-cervical intraepithelial neoplasia grade 3-carcinoma *in* *situ* (CIN3-CIS), invasive squamous cell carcinoma (SCC), adenocarcinoma *in* *situ* (AIS), and adenocarcinoma (ADCA) were referred here as CIN3 or worse (CIN3+). Squamous high-grade lesions included CIN3-CIS and SCC. Glandular high-grade lesions included AIS and ADCA. Invasive lesions included SCC and ADCA.

### HPV Detection and Genotyping

Two different storage media and two different tests were used for HPV detection and genotyping. Samples were maintained at 2–8°C and processed within 24–72 hours after collection. QIAamp MinElute Media Kit (Qiagen, Hilden, Germany) was used for DNA extraction. The extracted nucleic acids were stored at −20°C. An aliquot of the original sample was also stored at −20°C.

In the case of 138 patients, endocervical samples were collected in ThinPrep PreservCyt Solution (Cytic Corporation, Boxborough, MA, US). After HPV detection with AMPLICOR HPV detection kit (Roche Diagnostics, Mannheim, Germany), genotyping was carried out using the Linear Array HPV Genotyping Test (Roche Diagnostics, Mannheim, Germany) according to the manufacturer’s instructions.

In the case of 100 patients (including 83 additional cases), endocervical samples were collected in TE buffer pH 8.0 Molecular Biology grade (AppliChem GmbH, Darmstadt, Germany) and genotyping was carried out using the Linear Array HPV Genotyping Test according to the manufacturer’s instructions.

In the case of 158 patients (including 26 additional cases), endocervical samples were collected in TE buffer pH 8.0 Molecular Biology grade. Nested PCR was used to amplify the L1 conserved region as described previously [17]. Consensus oligonucleotides (MY09/MY11 as outer and GP5+/GP6+ as inner oligonucleotides) and FastStart Taq DNA polymerase (Roche Diagnostics, Mannheim, Germany) were used for amplification. HPV genotypes were identified by direct sequence analysis of the PCR products using the same oligonucleotides. The protocol is described below (see the (sub)lineages characterization section). Genotypes were assigned by alignments of HPV sequences (homology rate>90%) with those present in the GenBank database using the BLAST software (Basic Local Alignment Search Tool) (http://blast.ncbi.nlm.nih.gov/).

### (Sub)lineages Characterization Based on LCR/E6

HPV lineages were characterized in samples at enrolment. For their identification, 751 bp from the LCR-E6 region of HPV16 and 956 bp from the same region of HPV18 were amplified with the primers described previously [15]. The PCR mix contained 25 pmol of each primer, 0.2 mM of each deoxynucleotide triphosphate, 1x PCR buffer, 2 mM MgCl2, 1IU of Expand High Fidelity Taq DNA polymerase (Roche Diagnostics, Mannheim, Germany), and 5 µl of extracted sample in a 25 µl reaction volume. We performed PCR amplification with initial denaturalization at 94°C for 5 min followed by 45 cycles at 94°C for 30 sec, 55°C for 45 sec, and 72°C for 3 min and the final extension at 72°C for 10 min.

PCR products were purified with YM-100 PCR filter units (Millipore Corporation, Bedford, MA, USA) or PCR cleanup filter plates (Millipore Corporation, Temecula, CA, USA). DNA concentration was quantified on a NanoDrop ND-1000 spectrophotometer (Thermo Fisher Scientific, Wilmington, NC, USA) and adjusted to 20–40 ng/µl. Amplicons were automatically sequenced in a 5 µl reaction volume using the Sequencing BigDye Terminator v1.1 Cycle Sequencing Ready Reaction Kit (Applied Biosystems, Life Technologies Corporation, Austin, TX, USA) and then purified with the Montage 96-SEQ (Millipore Corporation, Temecula, CA, USA). All the primers used in amplification and sequencing reactions were listed in Table S1. Forward and reverse DNA sequences (two forward and two reverse in case of HPV18) were obtained with an ABI PRISM 3100 Avant Sequencer (Applied Biosystems, Life Technologies Corporation, Foster City, CA, USA). The electropherogram files were edited and assembled using Chromas Pro 1.5 (Technelysium Pty, Tewantin, Australia) and sequence variation was determined from both directions. SNPs were interpreted visually in comparison to the HPV16 (7906 bp) or HPV18 (7857 bp) prototype sequences previously described [18], [19] using a specific software online. The nucleotide sequence data were also examined using the BioEdit (version 7.0.5.3) sequence analysis program [20]. Multiple sequence alignments were carried out using the ClustalW program [21]. For phylogenetic analysis, a maximum likelihood (ML) tree was inferred from the alignments using RAxML HPC v8 [22]. FigTree 1.4 was used to view phylogenetic trees. We included in the analysis 10 LCR/E6 reference sequences (719 bp) in case of HPV16 analysis and nine LCR/E6 reference sequences (983 bp) in case of HPV18 analysis. HPV16 isolates were classified according to the lineages and sublineages described previously [4]. Within the D lineage, some HPV16 isolates could not be classified properly as D2 or D3 because 7743 position was not sequenced. HPV18 isolates were classified according to the lineages and sublineages described previously [4]. HPV sublineage prevalence was described for the different histological diagnoses. Prevalence of E-350G, E-350T variants or a mixture of both (E-350K) was also described for HPV16. Representative sequences from each group of highly related sequences (<0.4 differences) obtained in this study were deposited in GenBank (HPV16: KJ543714-28. HPV18: KJ543707-13).

### L1 Region Characterization

L1 region was characterized in samples at enrolment. A total of 423 bp from the L1 region of HPV16 were amplified by nested PCR with primers described previously (table S1) [23] including nucleotide triphosphate, 1x PCR buffer, 2 mM MgCl2, 1IU of Expand High Fidelity Taq DNA polymerase (Roche Diagnostics, Mannheim, Germany), and 5 µl of extracted sample in a 25 µl reaction volume. We performed PCR amplification with initial denaturalization at 95°C for 5 min, followed by 40 cycles at 95°C for 30 sec, 55°C for 30 sec, and 72°C for 50 sec and final extension at 72°C for 10 min for the first amplification, then at 94°C for 10 min, followed by 45 cycles at 94°C for 30 sec, 54°C for 45 sec, and 72°C for 3 min and final extension at 72°C for 10 min for the nested PCR.

PCR products were purified and sequenced with internal primers as described above (see the [Sub] lineages Characterization Based on LCR/E6 section). The prevalence of nucleotide changes in the studied region was described. Representative sequences from each group of highly related sequences (<0.4 differences) obtained in this study were deposited in GenBank (KJ549646-52).

### Statistical Analysis

The increased risk for CIN3+, squamous high-grade lesions, glandular high-grade lesions and invasive lesions associated with certain HPV lineages was calculated using Stata 12 (StataCorp LP, TX). *Odds ratio*s (OR) and corresponding 95% confidence intervals (95% CI) were estimated to assess the strength of association between HPV lineage and cervical lesion risk. Available epidemiological characteristics were described for different groups of HPV lineages. For all calculations of means, standard deviation was calculated. Means were compared using T-Student test. Qualitative variables were compared with the Fisher or the chi-square tests (SPSS version 15, Statistical Package for Social Sciences, Chicago, IL).

Statistical tests performed in the present study were considered significant whenever two-sided *p*-value was <0.05.

## Results

Lineage was established in 333 patients. A total of 217 cases and 116 controls were eligible for final analysis. Results of HPV persistence and progression at the end of the two-year follow-up of patients without histological lesion at enrolment are shown in table S2. Due to missing follow-up or treatment for CIN1, 29 women were excluded from the study.

Some epidemiological characteristics that may be related to cervical lesions risk are briefly described in table 1 for CIN3+ cases and control patients.

**Table 1:** Epidemiological characteristics of some patients included in the case-control study.

	HPV 16					HPV18			
	LINEAGE A		LINEAGE D			LINEAGE A		LINEAGE B	
	CONTROL	CIN3+	CONTROL	CIN3+	*p*	CONTROL	CIN3+	CONTROL	CIN3+
Age, y	35.6 (10.6, 82)	35.3 (9.0, 126)	26.5 (4.2, 4)	42.3 (13.5, 19)	*	31.6 (8.9, 23)	41.0 (10.9, 5)	21.0 (1)	28.1 (1.7, 2)
>30 years	51/82 (62.2)	85/126 (67.5)	1/4 (25)	17/19 (89.5)	*	11/23 (47.8)	4/5 (80)	0/1 (0)	0/2 (0)
HIV coinfection	0/74 (0)	3/68 (4.4)	0/4 (0)	1/10 (10)		0/19 (0)	0/3 (0)	-	0/2 (0)
Caucasian race	63/68 (92.6)	58/65 (89.2)	3/3 (100)	6/10 (60)		19/22 (86.4)	3/4 (75)	-	1/1 (100)
>5 years since FSI	65/74 (87.8)	66/68 (97.1)	3/4 (75)	11/11 (100)		18/22 (81.8)	4/4 (100)	-	1/1 (100)
>5 years use of HC	47/74 (63.5)	45/66 (68.2)	3/4 (75)	9/10 (90)		13/22 (59.1)	3/4 (75)	-	1/1 (100)
>5 years smoking	47/74 (63.5)	49/68 (72.1)	1/4 (25)	6/11 (54.5)		10/22 (45.4)	2/4 (50)	-	1/1 (100)
Current smoking	30/74 (40.5)	33/68 (48.5)	0/4 (0)	3/11 (27.3)		6/22 (27.3)	0/4 (0)	-	1/1 (100)

Age is given as mean (SD, n) and other as n/N (%).

FSI: First sexual intercourse.

HC: Hormonal contraceptives.

*p<0.05, statistically significant, control group as reference.

CIN3+ includes cervical intraepithelial neoplasia grade 3-carcinoma in situ, invasive squamous cell carcinoma, adenocarcinoma *in* *situ* and adenocarcinoma.

### Characterization of LCR/E6 Region of HPV16

**Figure 2:**
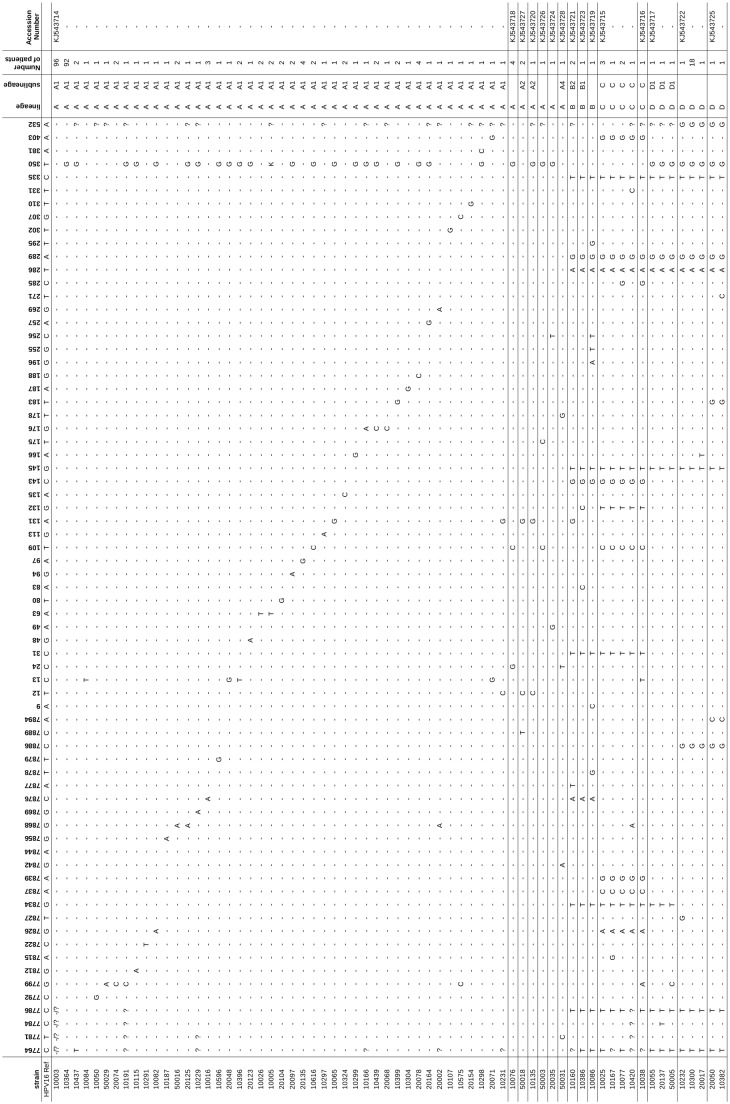
Nucleotide sequence variations of LCR/E6 among HPV16 isolates. Position number refers to the HPV 16 prototype sequence previously described [19].

**Figure 3:**
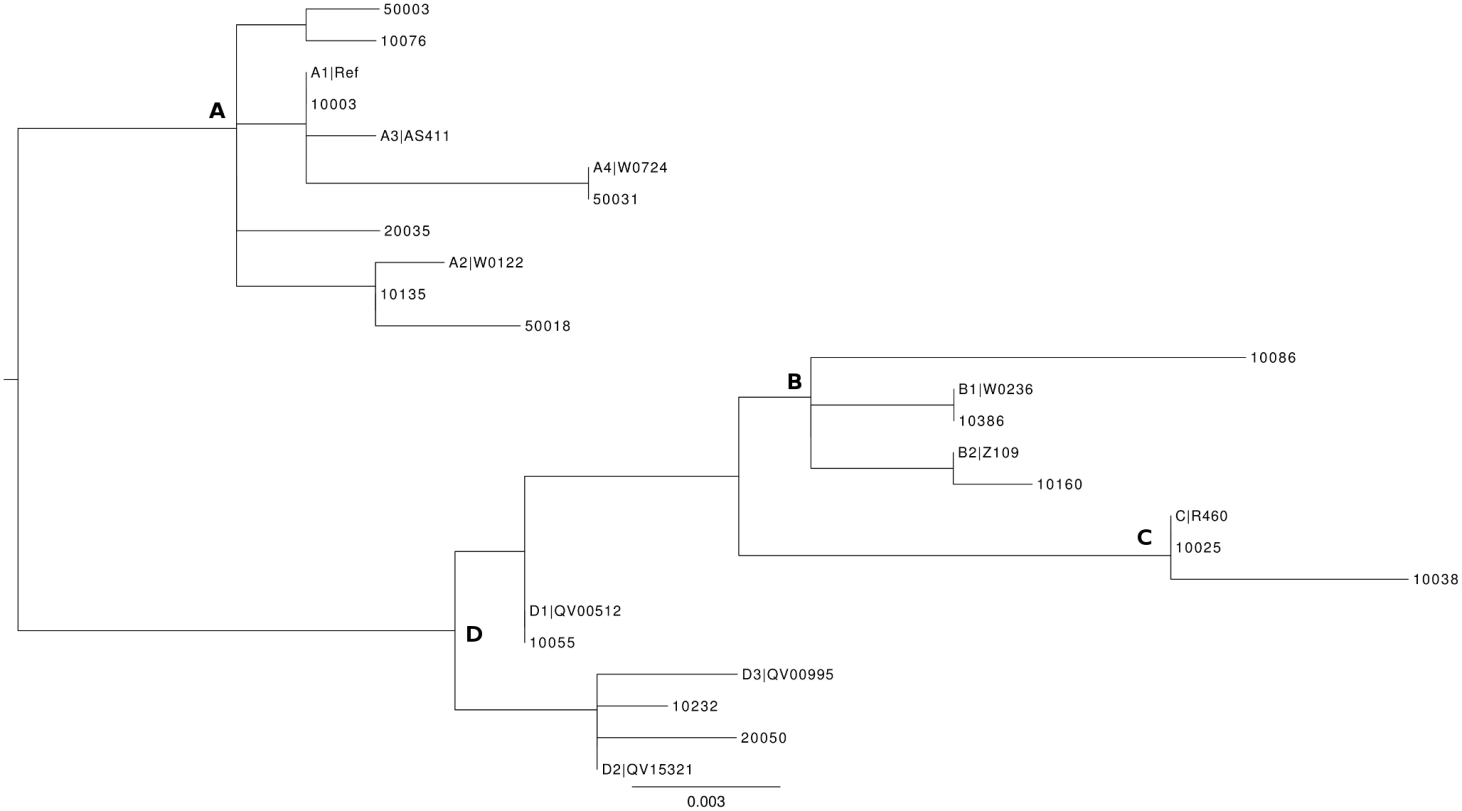
Phylogenetic tree of the HPV16 isolates based on LCR/E6 sequences. Phylogenetic analysis confirmed the presence of the four lineages [4]: A, B, C and D. A maximum likelihood (ML) tree was inferred from an alignment of ten reference sequences and fifteen study sequences of HPV16 LCR-E6 using RAxML HPC v8 [22]. Highly related sequences (<0.4 differences) from the study were not included in this figure. Reference sequences were denominated as lineage|strain.

The four lineages of HPV16 were found in the studied population (Fig. 2 and 3). HPV16 clustered in lineage A (260/297, 87.5%), lineage B (4/297, 1.3%), lineage C (8/297, 2.7%), and lineage D (25/297, 8.4%) (table 2). Prevalence of lineage D was 85.7% (6/7) in glandular high-grade lesions. Overall prevalence of the 350G polymorphism in European HPV16 was 134/259 (51.7%) (table S3).

**Table 2:** The distribution of lineages of HPV16 by cervical lesion group.

		HPV16 lineage n				D *vs* A lineage	
		A	B	C	D	Odds Ratio (95% CI)	*p*
Control		82	3	3	4	-	-
CIN2		52	-	-	2	-	-
CIN3+	All	126	1	5	19	3.1 (1.0–12.9)	0.04*
	CIN3-CIS	120	1	5	11	-	-
	SCC	5	-	-	2	-	-
	AIS	1	-	-	4	-	-
	ADCA	-	-	-	2	-	-
	CIN3-CIS and SCC	-	-	-	-	1.7 (0.5–7.6)	0.43
	AIS and ADCA	-	-	-	-	123 (9.7–5713.6)	<0.0001*
	SCC and ADCA	-	-	-	-	16.4 (2.2–113.7)	0.002*

CIN2: Cervical intraepithelial neoplasia grade 2. CIN3-CIS: CIN grade 3-carcinoma in situ. SCC: Invasive squamous cell carcinoma. AIS: Adenocarcinoma *in* *situ*. ADCA: Adenocarcinoma.

CIN3+ includes CIN3-CIS, SCC, AIS, and ADCA.

*Statistically significant, control group as reference.

Lineage D and lineage A of HPV16 were respectively detected in 4/82 control patients, 19/126 CIN3+ cases (OR = 3.1, 95%CI: 1.1–12.9, *p* = 0.04, control group as reference), 6/1 glandular high-grade lesions (OR = 123, 95%CI: 9.7–5713.6, *p*<0.0001), and 4/5 invasive lesions (OR = 16.4, 95%CI: 2.2–113.7, *p* = 0.002) (table 2).

Average age at the histological diagnosis of HPV-16 positive CIN3+ cases was 35.0±9.0 (n = 137) for CIN3-CIS, 46.0±18.0 (n = 7) for SCC, 44.8±12.0 (n = 5) for AIS, and 52.0±7.1 (n = 2) for ADCA. Patients with glandular high-grade lesions (n = 7) were older than those with squamous high-grade lesions (n = 144), (46.9±10.8 *vs.* 35.5±9.8, *p* = 0.003). HIV coinfection was detected in 6/198 (3%) HPV16- infected women (two patients diagnosed of CIN2 and four diagnosed of CIN3+).

### Characterization of LCR/E6 Region of HPV18

**Figure 4:**
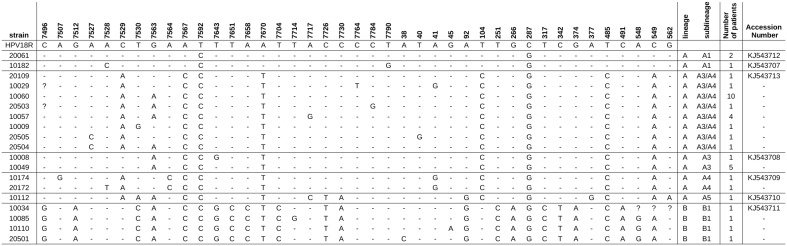
Nucleotide sequence variations of LCR/E6 among HPV18 isolates. Position number refers to the HPV18 prototype sequence previously described [18].

**Figure 5:**
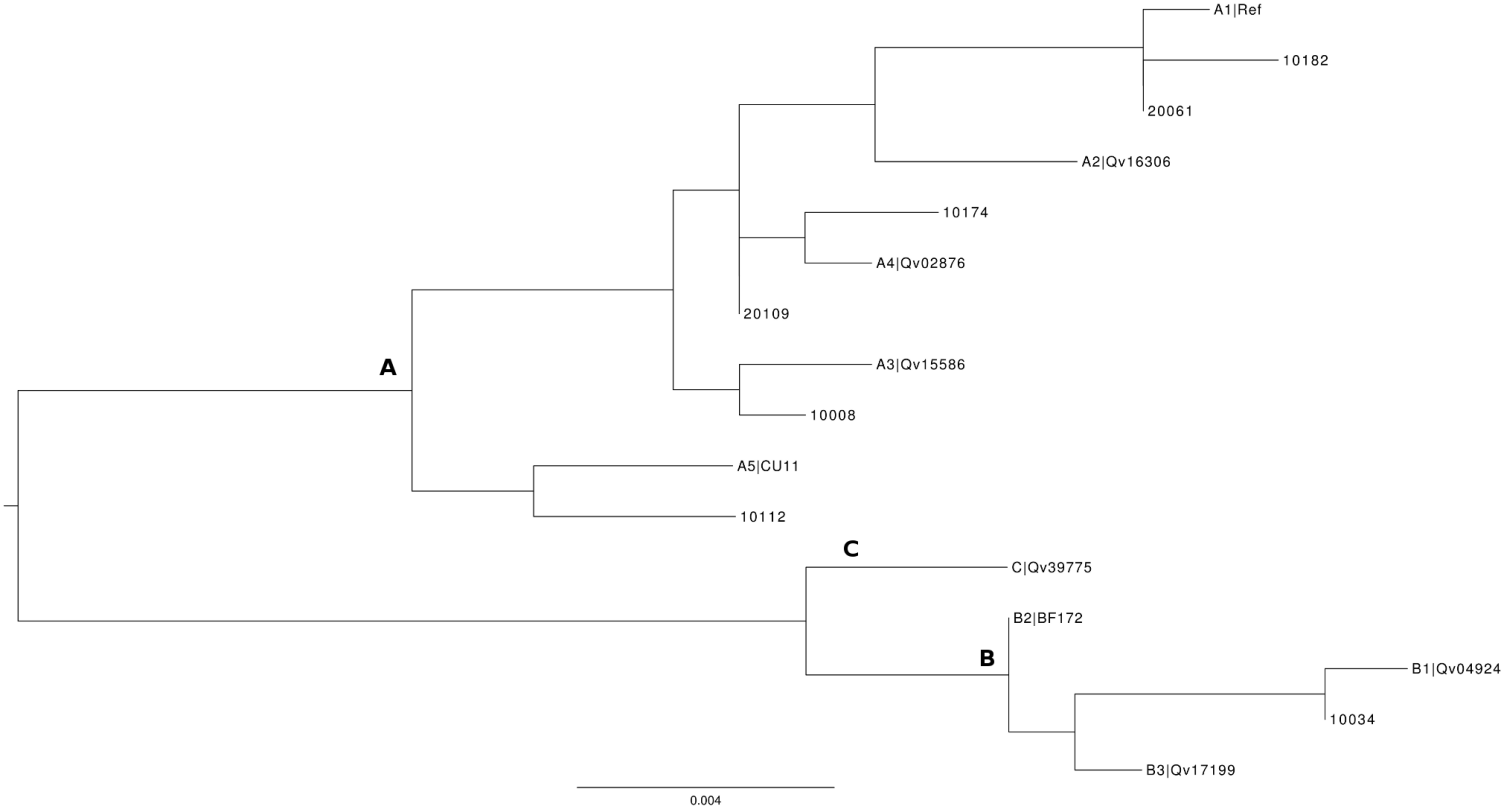
Phylogenetic tree of the HPV18 isolates based on LCR/E6 sequences. Phylogenetic analysis confirmed the presence of two lineages [4]: A and B. A maximum likelihood (ML) tree was inferred from an alignment of nine reference sequences and seven study sequences of HPV18 LCR-E6 using RAxML HPC v8 [22]. Highly related sequences (<0.4 differences) from the study were not included in this figure. Reference sequences were denominated as lineage|strain.

Two lineages previously described of HPV18 were found in the studied population (Fig. 4 and 5). HPV18 clustered in lineage A (32/36, 88.9%) and lineage B (4/36, 11.1%) (table 3). Lineage B and lineage A strains were respectively detected in 1/23 control patients and 2/5 CIN3+ cases (OR = 9.2, 95%CI: 0.4–565.4, p = 0.12, control group as reference) (table 3).

**Table 3:** The distribution of HPV18 lineages in the case-control study.

		HPV18 lineage n (%)		B *vs* A lineage	
		A	B	*Odds ratio* (95%CI)	*p*
Control		23	1	-	-
CIN2		4	1	-	-
CIN3+	All	5	2	9.2 (0.4–565.4)	0.12
	CIN3-CIS and SCC	3	1	-	-
	AIS and ADCA	2	1	-	-

CIN2: Cervical intraepithelial neoplasia grade 2. CIN3-CIS: CIN grade 3-carcinoma in situ. SCC: Invasive squamous cell carcinoma. AIS: Adenocarcinoma *in* *situ*. ADCA: Adenocarcinoma.

CIN3+ includes CIN3-CIS, SCC, AIS, and ADCA.

*p*, control group as reference.

### Characterization of L1 region of HPV16

**Figure 6:**
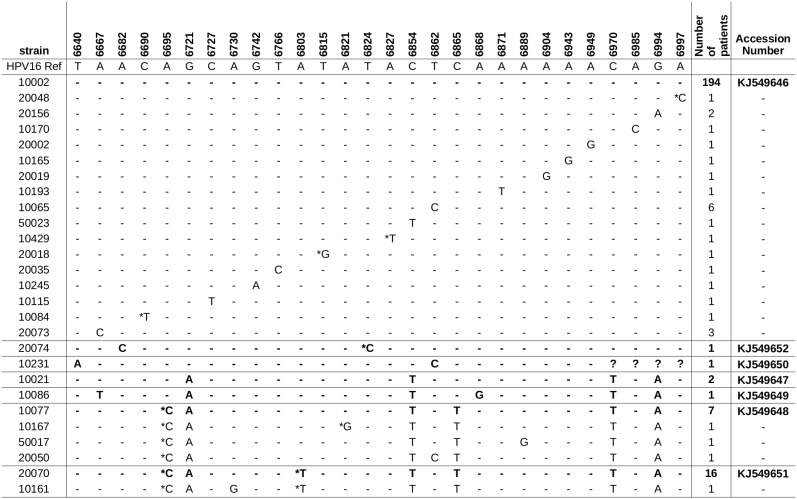
Nucleotide sequence variations of L1 among HPV16 isolates. Position number refers to the HPV 16 prototype sequence previously described. Asterisk indicates amino acid substitution.

Sequencing data of the capsid region were obtained for a total of 250 women infected by HPV16 included in the study, and with a valid follow-up. Nucleotide sequence variations of L1 among HPV16 isolates are shown in figure 6. Genetic variability analysis of L1 region revealed 21 silent nucleotide substitutions and eight nucleotide variations that lead to amino acid changes. The two more frequent non-synonymous SNPs were 6695C (27/250, 10.8%) and 6803T (17/250, 6.8%). The SNP 6803T was exclusively detected in strains previously characterized as lineage D by the LCR/E6 region analysis. The 6803A and 6803T strains were respectively found in 3/88 control patients, 5/1 glandular high-grade lesions (OR = 146.7, 95%CI: 9.9–6996.7, p<0.0001), and 4/4 invasive lesions (OR = 29.3, 95%CI: 3.4–254.6, p<0.001).

No deletions, insertions or recombinations were detected in the analyzed HPV16/18 regions. Mixed populations of HPV strains of the same lineage which differ in certain SNPs were detected in 5/333 (1.5%) patients. Coinfection by two different lineages of the same genotype was not detected in this study.

## Discussion

Specific regions of the HPV genome differ in their power to discriminate between all different lineages. In the present study, part of the LCR and E6 region of the HPV16 and HPV18 genome were used for (sub)lineage assignment as these fragments were found to be able to distinguish between all HPV16 and 18 sublineages [3], [24]. Four lineages of HPV16 and two lineages of HPV18 were detected in the studied population. The majority of HPV16/18 clustered in the corresponding lineage A, as expected in Spain [6], [8].

In patients with glandular high-grade lesions, the lineage D of HPV16 was the most commonly found, as previously reported by other authors [13], [25]. Lineage D was also detected in 8% of HPV16-positive squamous high-grade lesions.

Two lineages of HPV18 were found in both cases and control patients. These data differ from those found in another Spanish study [6] where the only lineage present in case of high-grade cytological lesions was the African lineage.

The distribution of HPV16 lineages around the world has been reported to be highly geographically/ethnically specific and their relative risk for cervical cancer has been suggested to be population dependent [8]. There is strong evidence that HPV16 NE sublineages have elevated risk for cancer. Specifically, lineage D has been previously associated with CIN3+ risk [7], [12], [26], [27]. Besides, it was observed recently [12] that there was a possible increased risk for CIN3+ for D2 sublineage (AA2). In the studied population, lineage D was associated with a risk for CIN3+3-fold higher than lineage A. Lineage D was also associated with a higher risk for invasive and glandular high-grade lesions than lineage A. Considering that the association of lineage D with an increased risk for squamous high-grade lesions compared with lineage A was not significant, the results discussed in this paper for HPV16-positive CIN3+ cases might be mainly influenced by the glandular and invasive lesions included in this histological group. The mean age at enrolment of CIN3+ cases was higher than the mean age of control patients in the lineage D group. This could be explained by the association of this lineage with the glandular lesions and the difficult diagnosis of these lesions. Patients with HPV16 infection presenting glandular high-grade lesions were older than those with squamous high-grade lesions (47 years old versus 35 years old).

For HPV16 data analysis, we combined the CIN3+ cases from a prospective study and the CIN3+ supplemental cases drawn from the same population at a different period of time for increased analytic power. Differences in mean age, proportion of CIN3, or glandular high-grade lesions were not significant. Besides, lineage D was similarly distributed between prospective study cases and supplemental cases, justifying combining the two groups.

The common 350G polymorphism is a no lineage-specific SNP localized in the E6 oncogene. It was studied within the European sublineages of HPV16, but no differences were observed in the prevalence of this SNP in relation with the presence of cervical lesions or the age of the patients. Previously reported differences [28] in age might be related with an uneven distribution of invasive lesions between the compared groups.

With regard to HPV18, there is a lack of evidence of the lineages’ role in cancer pathogenesis. Although it is the second most common HPV in cervical cancer after HPV16, it is much less prevalent, and the precancerous lesions are infrequently detected, so progression studies are not easy to perform. Variation at nucleotide 104 was associated to a higher activity of the E6/E7 promoter [29]. AA sublineages (A1, A2) of HPV18 could have increased ability of inducing tumor formation in vivo [30]. Some authors reported a greater risk of lineage A [31]. In this study, lineage B (104T) may be associated with increased risk for CIN3+ compared with lineage A, but the association was not significant, probably due to the small number of included patients. The lineage analysis of the different regions (LCR, E6) was concordant, so the isolates that we have studied did not appear to be the product of genetic recombination between viruses of different lineages. Therefore, in order to simplify the methodology, a single fragment that gives enough information could be used in further lineage studies. Multiple HPV16 variants in one woman were reported to be rare (8.6%) [32], but the prevalence is even lower in this study. The observed difference may be due to differences in the sensitivity and specificity of the methods used to identify variants. Sanger sequence analysis is an insensitive method to detect minority variants, with a sensitivity of approximately 25% for minority populations.

HIV prevalence was 3% in HPV16-infected women. Given the relationship between HIV and HPV in cervical cancer, it might be interesting to take into consideration the HIV coinfection in future studies of cervical cancer risk of HPV lineages.

The study of the L1 region individually provides limited information on viral lineage but it could be helpful if specific changes in this region were related with severe disease. A part of HPV16 L1 region, commonly used for the detection of HR-HPV in clinical specimens, was sequenced in a limited number of patients previously characterized by LCR/E6 sequencing. In this study, eight amino acid substitutions were identified. The two more common non-synonymous SNPs found in the L1 region were 6695C and 6803T. The 6695C SNP leads to the replacement of Thr at position 353 by Pro. The 6803T polymorphism that results in a substitution of Thr by Ser at position 389 was present in samples that clustered in lineage D in this study. The detection of the 6803T polymorphism was associated to an increased risk for high-grade glandular and invasive lesion compared with the detection of 6803A. It may be interesting to conduct further studies to investigate the usefulness of this specific SNP detection using quicker and cheaper methods than nucleic acid sequencing analysis. The incorporation of a lineage-specific SNP detection to the current L1-based genotyping methods could help to get a deeper knowledge of the CIN3+ risk of HPV16.

Understanding the genetic basis of the special carcinogenicity of some HPV16 and HPV18 lineages may help us to discover interactions between the virus and the host that could be important to achieve a better control of HPV infection and cervical cancer. Few epidemiological studies of HPV lineages referring to the Spanish population have been published so far [6], [28], [33]–[35] and only one of them refers to HPV18. It was observed in a recent multicentre case-control study that the distribution of HPV16 lineages worldwide and their relative risks for cervical cancer could be population-dependent [8], so it seems interesting to provide data from a specific geographical region.

One limitation of the present study is the sample size that restricted the analysis of some histological types of cervical lesions and the analysis of some lineages not commonly found in Europe. Different methods were used for HR-HPV genotyping; this could lead to differences in HPV16/18 detection. Another limitation is the two-year follow-up because some patients included in the control group could have progressed in a longer follow-up. It would be interesting to perform collaborative studies including a larger number of patients to confirm the obtained results.

In summary, lineage A of HPV16 was predominant in Spanish women. Lineage D was associated with increased risk for CIN3+ compared with lineage A. Further large well-designed, age-adjusted studies are needed before the application of HPV16 lineage detection as a high-grade lesion biomarker in clinical settings.

## Supporting Information

Table S1 **Primers for characterization of HPV16/18 LCR/E6 region and HPV16 L1 region.**
(DOC)

Table S2 **Two-year follow-up of women without cytological lesions at enrolment.** The two-year follow-up of women that were attending the Gynecology and Obstetrics Department for cervical cancer screening. Virological and cytological control every six months to assess viral persistence and progression to moderate or high-grade cervical lesions. ASC-US (atypical squamous cells of undetermined significance), LSIL (low-grade squamous intraepithelial lesion), HSIL (high-grade squamous intraepithelial lesion), ASC-H (atypical squamous cells, it is not possible to exclude HSIL). Persistence: Virological persistence. Progression: CIN2+ at enrolment or progression to CIN2+ in follow-up.
(DOC)

Table S3 **Frequency of SNP 350G of European (E) HPV16.** CIN2: Cervical intraepithelial neoplasia grade 2. CIN3-CIS: CIN grade 3-carcinoma in situ. SCC: Invasive squamous cell carcinoma. AIS: Adenocarcinoma *in* *situ*. ADCA: Adenocarcinoma. CIN3+ includes CIN3-CIS, SCC, AIS, and ADCA. 350K: mix of 350T and 350G.
(DOC)
