# Supplementary material for: Prevalence of HPV 16 and HPV 18 Lineages in Galicia, Spain
Source: PLoS One. 2014 Aug 11;9(8):e104678. doi: 10.1371/journal.pone.0104678 (PMC4128731; doi:10.1371/journal.pone.0104678)
Supplement: Table S1 — Primers for characterization of HPV16/18 LCR/E6 region and HPV16 L1 region. (DOC) [file pone.0104678.s001.doc]

**Table S1: Primers for characterization of HPV16/18 LCR/E6 region and HPV16 L1 region**

| **DNA REGION** | **GENOTYPE** | **CHARACTERISTICS** | **SENSE AND ANTISENSE PRIMERS** | **USE** |
| --- | --- | --- | --- | --- |
| LCR/E6 | HPV16 | Forward | 5′-AGGCACATATTTTTGGCTTGTT-3′ | Amplification and sequencing |
|  |  | Reverse | 5′-TTCATGCAATGTAGGTGTATCTCC-3′ | Amplification and sequencing |
|  | HPV18 | Forward | 5′-GTTGCCTTTGGCTTATGTCTG-3′ | Amplification and sequencing |
|  |  | Reverse | 5′-TTGCCTTAGGTCCATGCATAC-3′ | Amplification and sequencing |
|  |  | Forward, inner primer | 5′-AATACTATGGCGCGCTTTGA-3′ | Sequencing |
|  |  | Reverse, inner primer | 5′-TGTCTTGCAGTGAAGTGTTCAG-3′ | Sequencing |
| L1 | HPV16 | Forward, outer primer | 5′-GCMCAGGGWCATAAYAATGG-3′ | Amplification |
|  |  | Reverse, outer primer | 5′-CGTCCMARRGGAWACTGATC-3′ | Amplification |
|  |  | Forward, inner primer | 5′-AATGGCATTTGTTGGGGTA-3′ | Amplification and sequencing |
|  |  | Forward, inner primer | 5′-TAATGGCATTTGTTGGGGT-3′ | Amplification and sequencing |
|  |  | Reverse, inner primer | 5′-CTGATCTAGGTCTGCAGAAAAC-3′ | Amplification and sequencing |
