# Supplementary material for: Prevalence of HPV 16 and HPV 18 Lineages in Galicia, Spain
Source: PLoS One. 2014 Aug 11;9(8):e104678. doi: 10.1371/journal.pone.0104678 (PMC4128731; doi:10.1371/journal.pone.0104678)
Supplement: Table S2 — Two-year follow-up of women without cytological lesions at enrolment. The two-year follow-up of women that were attending the Gynecology and Obstetrics Department for cervical cancer screening. Virological and cytological control every six months to assess viral persistence and progression to moderate or high-grade cervical lesions. ASC-US (atypical squamous cells of undetermined significance), LSIL (low-grade squamous intraepithelial lesion), HSIL (high-grade squamous intraepithelial lesion), ASC-H (atypical squamous cells, it is not possible to exclude HSIL). Persistence: Virological persistence. Progression: CIN2+ at enrolment or progression to CIN2+ in follow-up. (DOC) [file pone.0104678.s002.doc]

**Table S2: Two-year follow-up of women without cytological lesions at enrolment**

|  | HPV 16 n (%) | | | HPV 18 n (%) | |  |
| --- | --- | --- | --- | --- | --- | --- |
|  | No persistence | Persistence  No progression | Progression | No persistence | Persistence  No progression | Progression |
| HSIL/ASC-H | 5 (9.8) | - | 46 (90.2) | - | - | 4 (100) |
| LSIL | 14 (29.8) | 7 (14.9) | 26 (55.3) | 10 (71.4) | 2 (14.3) | 2 (14.3) |
| ASC-US | 16 (42.1) | 7 (18.4) | 15 (39.5) | 6 (60) | 1 (10) | 3 (30) |
| No lesions | 38 (52.8) | 20 (27.8) | 14 (19.4) | 14 (63.6) | 3 (13.6) | 5 (22.7) |

The two-year follow-up of women that were attending the Gynecology and Obstetrics Department for cervical cancer screening. Virological and cytological control every six months to assess viral persistence and progression to moderate or high-grade cervical lesions.

ASC-US (atypical squamous cells of undetermined significance), LSIL (low-grade squamous intraepithelial lesion), HSIL (high-grade squamous intraepithelial lesion), ASC-H (atypical squamous cells, it is not possible to exclude HSIL).

Persistence: Virological persistence. Progression: CIN2+ at enrolment or progression to CIN2+ in follow-up
