# Supplementary material for: Prevalence of HPV 16 and HPV 18 Lineages in Galicia, Spain
Source: PLoS One. 2014 Aug 11;9(8):e104678. doi: 10.1371/journal.pone.0104678 (PMC4128731; doi:10.1371/journal.pone.0104678)
Supplement: Table S3 — Frequency of SNP 350G of European (E) HPV16. CIN2: Cervical intraepithelial neoplasia grade 2. CIN3-CIS: CIN grade 3-carcinoma in situ. SCC: Invasive squamous cell carcinoma. AIS: Adenocarcinoma in situ. ADCA: Adenocarcinoma. CIN3+ includes CIN3-CIS, SCC, AIS, and ADCA. 350K: mix of 350T and 350G. (DOC) [file pone.0104678.s003.doc]

**Table S3: Frequency of SNP 350G of European (E) HPV16**

|  | E-350T n (%) | E-350G n (%) | E-350K n (%) |
| --- | --- | --- | --- |
| Control | 39 (47.6) | 42 (51.2) | 1 (1.2) |
| CIN2 | 27 (52.9) | 23 (45.1) | 1 (2.0) |
| CIN3-CIS and SCC | 58 (46.4) | 66 (52.8) | 1 (0.8) |
| AIS and ADCA | 1 (100) | - | - |

**350K: mix of 350T and 350G**
